# Large Animal Models for Preclinical Evaluation of Heart Valve Prostheses, Left Ventricular Assist Devices and Total Artificial Hearts: A Narrative Review

**DOI:** 10.3390/biomimetics11040258

**Published:** 2026-04-08

**Authors:** Oskar Gülcher, Celeste Koster, Jolanda Kluin, Paul Gründeman

**Affiliations:** Department of Cardiothoracic Surgery, Thorax Center, Erasmus MC, 3015 GD Rotterdam, The Netherlands

**Keywords:** large animal models, preclinical evaluation, mechanical cardiac implants, prosthetic heart valves, ventricular assist devices, total artificial heart, translational research, calcification, thrombogenicity, hemodynamic performance, interspecies differences, animal model selection

## Abstract

Large animal models are a critical component of the preclinical evaluation of mechanical cardiac implants, enabling assessment of safety and performance under physiological conditions that cannot be adequately reproduced in vitro. Choosing a suitable animal model is important for both scientifically valid and ethically responsible preclinical evaluation. However, interspecies differences between animal models and humans pose significant challenges for relevant translation of preclinical findings to clinical outcomes. This narrative review provides a comprehensive overview of commonly used large animal models (sheep, goats, pigs, and calves) for the preclinical assessment of mechanical cardiac implants, including prosthetic heart valves, ventricular assist devices, and total artificial hearts. We summarize key anatomical and physiological characteristics that influence device implantation, chronic follow-up, and translational value. Emphasis is placed on three critical outcome domains for preclinical evaluation of mechanical cardiac implants: calcification, thrombogenicity, and hemodynamic performance. Species- and age-dependent differences in calcification are reviewed, identifying juvenile sheep as a worst-case model for early manifestation and detection of graft mineralization. Interspecies differences in coagulation biology are examined, showing attenuated platelet responses in sheep and closer similarity between porcine and human platelet behavior, supporting pigs as the preferred thrombogenicity model. Hemodynamic evaluation strategies in acute and chronic large-animal studies are discussed, with particular emphasis on circulatory demands influenced by somatic growth and on device adaptability under varying loading conditions. Overall, this review provides practical, outcome-driven guidance for large animal model selection and experimental design in mechanical cardiac implant research, while identifying key limitations, knowledge gaps, and the need for standardized reporting to improve the translational reliability of preclinical studies. Based on the findings presented in this review, we conclude that there is no single animal model capable of evaluating all relevant aspects of a device. Instead, different animal models provide distinct advantages depending on the outcomes of interest.

## 1. Introduction

To date, heart disease remains one of the most significant burdens on healthcare systems, affecting millions of people worldwide [1,2]. At advanced stages, when pharmacological therapy is no longer effective, heart disease may be managed surgically. This often involves the implantation of mechanical devices designed to replace or augment impaired cardiac function. Broadly, two key mechanical functions of the heart can be mimicked using implantable devices: the valvular and the pumping functions. Heart valve dysfunction is often treated by replacing a diseased valve with a valvular prosthesis. In cases of severely impaired myocardial contractility, the blood circulation can be mechanically supported through the implantation of a pump. This may involve supporting the function of the native heart—most commonly the left ventricle—using a ventricular assist device (LVAD), or complete replacement of the native heart by implantation of a total artificial heart (TAH). Although the latter procedure is currently performed only rarely, recent technological advances suggest potential for expanded clinical use of total artificial hearts in the future [3]. In this paper, prosthetic valves, LVADs, and TAHs are collectively referred to as mechanical cardiac implants, reflecting their function as devices with moving components that mechanically interact with the cardiovascular system.

Mechanical cardiac implants should be designed using biomimetic principles, with the aim of replicating key functional characteristics of the native heart’s biology. Although this approach involves a wide range of considerations, three essential properties are particularly critical for ensuring safe and durable long-term performance. These include decreased susceptibility to calcification, minimal thrombogenic risk, and efficient hemodynamic performance. Accordingly, these characteristics must be rigorously evaluated and validated throughout the development process and following clinical implementation.

The development of mechanical cardiac implants from initial concept to clinical application follows a structured trajectory comprising three main phases: preclinical studies, clinical studies, and post-market monitoring. The primary objective of the preclinical phase is to generate robust scientific evidence demonstrating that the device is safe and performs effectively for its intended clinical purpose. Only when this evidence has been provided is regulatory approval granted to advance to clinical studies [4]. A framework for conducting preclinical assessments is provided by ISO (International Organization of Standardization) guidelines, which consist of in vitro and in vivo components. During the in vitro phase, devices undergo extensive bench testing to verify mechanical performance, durability and, to some extent, biocompatibility under controlled conditions. Once established criteria are met, mechanical cardiac implants proceed to in vivo evaluation using animal models. Such evaluations typically focus on the key biomimetic properties outlined above, including resistance to calcification, low thrombogenic potential, and efficient hemodynamic performance. Some of these properties can, to a limited extent, be investigated using small animal models, typically rodents. However, because of their size constraints, small animals are not suitable for comprehensive testing of human-sized mechanical cardiac implants. Large animal models are required for in vivo evaluation of fully functional devices under physiological and mechanical conditions that closely resemble those of the human body.

Different large animal species can be used as models for humans in the preclinical evaluation of mechanical cardiac implants. Although non-human primates have historically been used extensively in translational surgical research, their application in the preclinical evaluation of mechanical cardiac implants has been limited. Instead, dogs served as the primary species for such investigations. For instance, the earliest heart valves and total artificial hearts were implanted in canine models [5]. The preference for dogs was largely practical: they were readily available, relatively easy to handle, and of suitable size for a wide range of experimental procedures [5]. However, increasing ethical concerns prompted a shift toward alternative large animal models [4,6]. As a result, sheep, goats, pigs, and calves have become the predominant species used in modern cardiac device research [7].

These animal species each possess distinct characteristics, and selecting the appropriate model for a specific research objective is therefore critical to ensuring the translational relevance and quality of experimental findings. However, at present, no general consensus exists regarding animal model selection, and ISO guidelines give no explicit recommendations [4]. The aim of this paper is to provide an overview of large animal models and experimental designs commonly employed in the preclinical evaluation of mechanical cardiac implants. We highlight the key advantages and limitations of animal models to support informed decision-making in animal model selection for preclinical studies. Specifically, we address relevant characteristics of sheep, goat, pig, and calf models with respect to device implantation, evaluation and clinical translation. Since the choice of animal models is closely linked to the outcomes of interest, these outcomes are used as a framework for the comparative discussion of the different models.

## 2. General Considerations in Animal Model Selection

**Figure 1:**
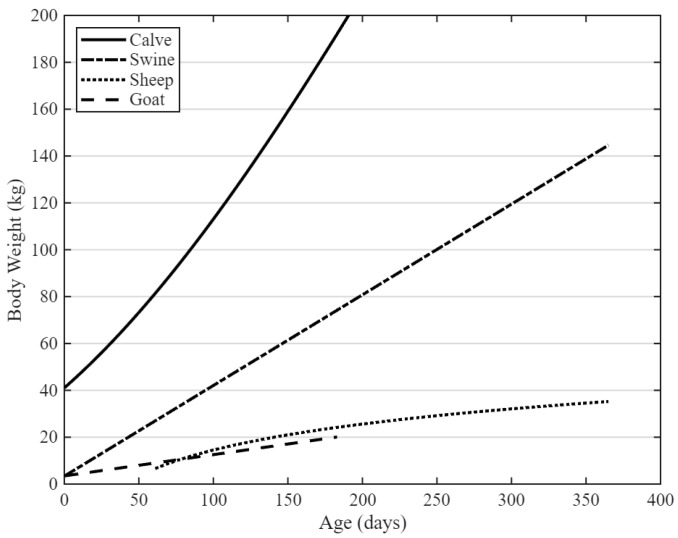
Growth curves of sheep, goats, pigs, and calves during their first year of life.

When planning large animal experiments for preclinical device evaluation, a solid understanding of the characteristics of commonly used large animal models, and their differences, is essential. Relevant general characteristics include body weight, growth patterns, and behavior, noting that substantial weight variation exists among breeds within a given species. Figure 1 illustrates the typical growth curves of sheep, goats, pigs, and calves during their first year of life [8,9]. From this figure, it is evident that pigs and calves outgrow typical human dimensions within this year. Sheep and goats reach their full body size only after approximately 24 months. At an adult age, goats typically weigh between 40.8 kg and 81.6 kg, while sheep range between 40.8 kg and 90.7 kg [8,9]. It is important to consider the somatic growth of animals when conducting chronic animal trials with long follow-up periods. As animals grow, implanted devices may become poorly anatomically fitted, leading to compromised experimental outcomes and reduced clinical translatability. Larger animals also require more resources and can be more difficult to handle, which adds logistical challenges. To mitigate these growth-related limitations associated with pig models specifically, minipigs have been selectively bred as an alternative. Their reduced growth rate makes them suitable for certain types of cardiac device research, such as prosthetic heart valve evaluation [10]. However, for LVAD and TAH evaluation, their limited anatomical dimensions are insufficient.

In contrast to acute animal experiments, which are limited to the surgical procedure performed under general anesthesia and followed by euthanasia, chronic animal experiments include a post-procedural follow-up period. During this period, the animal recovers from surgery and is housed in an animal housing facility. From an ethical perspective, animal welfare should be facilitated as much as possible throughout the follow-up. In addition, appropriate welfare conditions improve experimental reliability and minimize variability in results [11]. For effective animal welfare management and monitoring, an understanding of species-specific behavior and needs is essential.

Sheep, goats, calves, and pigs are social animals and should be housed in groups whenever possible, as this can promote positive behavioral interactions [12]. However, group housing of sexually intact males and females may also provoke aggression and mounting behavior, leading to injuries and chronic fear [13,14]. Therefore, female animals are typically used, as they are easier to handle and can be housed in groups [12]. If individual housing is required, a companion animal should be housed nearby to allow visual and preferably nose-to-nose contact [15,16].

Sheep and goats are small ruminants and, therefore, relatively easy to handle [15,17]. Compared to sheep, goats are more exploratory in nature and tend to chew on surrounding materials. This can present a particular challenge in LVAD or TAH research, where a percutaneous driveline is present. To prevent goats from chewing on external components, they are often housed in small restraining cages with limited head mobility during chronic studies involving percutaneous drivelines. Alternatively, the use of a cervical collar to prevent lateral bending of the neck has been successfully implemented in a previous study [18]. This setup allows the goat to move freely within the cage while preventing access to external components, thereby improving animal welfare.

Calves and pigs are larger and stronger animals, which can make them more difficult to handle during chronic studies. In addition, these animals require more space for housing and may continue to grow throughout the follow-up period, increasing housing requirements over time. Pigs may also exhibit more active or instinctive behaviors, such as rooting or wallowing, which may pose additional challenges in the presence of externalized components such as percutaneous drivelines [16,19].

## 3. Anatomical and Surgical Considerations

Differences between human and sheep, goat, pig and calf anatomy generally necessitate a different surgical approach. In humans, mechanical cardiac devices are typically implanted via sternotomy while cardiac procedures in large animal models are commonly performed through thoracotomy. Unlike the human thorax, which is compressed ventrodorsally, the thorax of quadruped mammals is compressed laterally [20]. Consequently, the heart and great vessels are oriented differently and are positioned relatively deep within the thoracic cavity. As a result, these structures are more readily accessible via thoracotomy than via sternotomy in large animal models [21]. A lower risk of wound complications and the relatively narrow sternum in sheep have also been reported as reasons to favor thoracotomy over sternotomy [21,22]. The increased risk of wound complications following sternotomy is likely attributable to the continuous pressure that is exerted on the sternum of quadrupeds in their natural resting position. Additionally, the prolonged unnatural dorsal recumbency required for sternotomy can result in compression of visceral organs and the inferior vena cava, causing venous congestion [23]. Regarding TAH implantation, however, a median sternotomy provides wider visualization of the anastomotic suture lines after connecting the device to the inflow and outflow ports [23,24].

In ruminants, the large fermentative forestomach poses a significant anesthetic challenge. Impaired eructation during general anesthesia leads to ruminal gas accumulation, causing diaphragmatic compression, impaired venous return, and thus compromised cardiopulmonary function. Therefore, preoperative fasting and intraoperative rumen decompression, typically via placement of a naso-ruminal tube, are essential for the safe execution of prolonged surgical procedures such as cardiac device implantation [25,26,27]. In contrast, pigs, as monogastric animals, do not experience these rumen-related limitations.

The relevance of anatomical similarities and differences between humans and animal models depends on the type of mechanical cardiac implant. For example, sheep are commonly used models in heart valve research. Their valvular architecture shows high resemblance to that of humans [28]. Unlike humans, sheep lack the membranous septum that provides fibrous continuity between the mitral and aortic valves, a structure closely associated with the bundle of His. In humans, its proximity to the conduction system makes transcatheter aortic valve implantation susceptible to conduction disturbances when valve positioning is suboptimal [29,30]. Because this septal component is absent in sheep, both the mechanism and risk of such conduction disturbances may differ from those observed in humans. Additionally, the absence of a calcified, stenotic aortic annulus can limit adequate transcatheter aortic valve prosthesis anchoring, resulting in dislocation of the device. To address this limitation, aortic banding is often performed in a separate prior procedure to reinforce the aortic annulus and facilitate adequate fixation [31,32].

**Figure 2:**
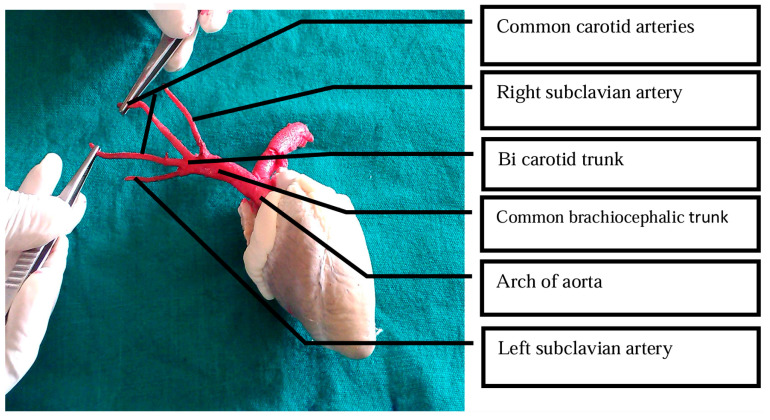
Aorta and its side branches in the goat heart. The ascending aorta is not visible due to its short length. From Shakuntala Rao et al. [35], used with permission from the authors.

For LVAD implantation, a key anatomical difference between humans and ruminants such as sheep, goats, and calves is the short ascending aorta (Figure 2) [33,34,35]. This anatomy precludes standard outflow graft anastomosis to the ascending aorta, necessitating attachment to the descending aorta instead [36]. More generally, the limited length of the ascending aorta also restricts surgical manipulation and complicates cannulation for cardiopulmonary bypass [37].

For TAH implantation, thoracic cavity size is often a decisive factor in animal model selection as TAHs tend to be relatively large devices. For chronic evaluation, the device must fit adequately within the thoracic cavity. An overly tight placement can cause compression of adjacent structures, such as the inferior vena cava, leading to complications [38]. In practice, TAHs frequently fit only within the thoracic cavity of calves, thereby limiting the suitability of other animal models for chronic experiments [23]. Therefore, despite their somatic growth, calves are commonly used as a model for chronic TAH evaluation. Nevertheless, full-grown sheep and goat models have also been used for chronic evaluation of smaller TAH designs and offer the advantage of no somatic growth throughout the experiment [38,39]. Imachi et al. reported on the dimensional requirements of their TAH to fit a goat model; by reducing the dimensions of their cylindrically shaped TAH to a length of 90 mm and an outer diameter of 80 mm, they achieved a suitable anatomical fit [39].

Pigs are less common models for chronic evaluation of mechanical cardiac implants despite offering certain benefits over ruminants. Compared to ruminants, the cardiac anatomy of pigs is generally considered the most similar to that of humans, particularly with respect to coronary anatomy [20,40]. Consequently, pigs are widely used for surgical training, acute experiments, and chronic evaluation of cardiovascular devices for which human-like coronary anatomy is considered relevant [41,42]. However, their suitability for long-term studies involving mechanical cardiac implants is limited by continuous growth, challenging handling and behavioral characteristics, and a higher susceptibility to postoperative infections [43].

## 4. Species and Age-Dependent Variations in Cardiac Device Calcification

Calcification is an important complication that occurs in certain mechanical cardiac implants made from synthetic and biological materials and can ultimately compromise device performance. This is a well-known issue in biological heart valves, but it has also been observed in some polymeric or tissue-engineered valve designs. Calcification may present a potential long-term concern for membrane-based blood pumps such as Carmat’s Aeson^®^ TAH, which utilizes bovine pericardium membranes, with ongoing research focusing on strategies to mitigate this risk [44,45]. Over time, calcification can cause structural deformations such as valve leaflet or membrane stiffening, loss of compliance, thinning, abrasion, degeneration, deformation, perforation, and embolization, compromising mechanical function and ultimately leading to device failure [46,47,48].

Calcification results from calcium phosphate deposition within or on biomaterials. This can develop intrinsically as mineralization takes place inside the material, extrinsically in association with attached cells, proteins, thrombi, or vegetations, or through combination of both. These mineralization processes typically arise from complex implant and host interactions and progress gradually over a period of weeks to months following implantation [46].

Both biological and synthetic materials can exhibit these calcification patterns, although the underlying mechanisms differ [49]. In tissue-derived materials, calcification is primarily influenced by factors such as chemical crosslinking with fixatives such as glutaraldehyde, cellular damage, extracellular matrix formation, host metabolic activity and immune responses, and cyclic mechanical loading and fatigue [47,50]. In contrast, calcification in synthetic materials mainly results from surface characteristics, including material morphology, porosity, and the presence of macro- and microdefects, which affect protein adsorption and subsequent cellular interactions [46,48].

Despite substantial progress in developing in vitro models to assess the susceptibility to calcification in cardiac devices, the complex interplay among biological, structural, and mechanical factors cannot be fully replicated in vitro or in small animal models [51,52]. Moreover, phenotypic and functional differences between rodent and human stem cells, which play a key role in calcification processes at cellular and molecular levels, may further limit the translational relevance of small animal studies [53,54,55,56]. Therefore, evaluating calcification risk, progression, and severity in cardiac devices requires implantation and long-term follow-up, extending over several weeks to months, in large animal models that more closely mimic human physiology [57].

Sheep are generally considered a highly suitable large animal model for investigating the occurrence of calcification in vivo, due to a quick tendency towards calcification compared to humans [47,58]. However, the theoretical basis for this assumption is not well established in the literature, with limited systematic comparisons of calcification progression across different animal species. Trantina-Yates et al. compared calcification progression in xenograft valved aortic roots implanted in sheep versus baboons, as primates generally share more similarities with human cardiac anatomy and physiology than non-primate species [59]. After 6 weeks, calcium content of explanted grafts was analyzed using atomic absorption spectroscopy. Leaflet calcification remained minimal and did not differ considerably between species. However, calcium content in the graft wall was significantly higher in sheep (64.8 ± 39.8 µg/mg) than in baboons (4.1 ± 5.9 µg/mg; *p* < 0.005). These findings indicate the generally accelerated mineralization observed in sheep compared to humans, supporting the use of ovine models as a worst-case scenario for evaluating calcification.

In the context of tissue-engineered heart valves, the pronounced tendency for calcification in the ovine model may present important limitations [60]. Functional performance of these valves depends on a delicate balance between scaffold degradation and new tissue formation [61]. The accelerated calcification observed in sheep may disrupt this balance and potentially distort outcomes. However, the influence of calcification on tissue engineering dynamics remains unclear. Notably, however, a systematic review assessing calcification in tissue-engineered pulmonary heart valves reported no significant differences between porcine and ovine models [62].

Importantly, juvenile animals demonstrate a substantially higher rate of calcification than adults in response to implanted materials. This is also observed in clinical practice as younger patients often undergo more rapid calcium-induced structural degeneration [50,63,64]. The age-related differences in calcification rates can largely be explained by more active immune and inflammatory responses in younger individuals [65]. Schoen et al. observed that bioprosthetic valves implanted in mitral position in juvenile sheep (3–4 months old) demonstrated early onset and rapid progression of calcification [66]. Calcific deposits were found as early as 23 days in the aortic wall and 31 days in the valve cusps. These results align with results obtained by Flameng et al. where ovine age significantly influenced the calcification rate [63]. Sheep age (5 versus 11 months) was shown to be an independent factor of bioprosthetic valve calcification. Thus, juvenile sheep have a strong tendency for calcification and are valuable models for early detection of valve mineralization. However, their growth potential may cause patient-prosthesis mismatch during long-term follow-up.

Blood pump calcification in membrane-based LVADs and TAHs only became a problem after improved device durability led to extended animal survival [67]. Mineralization of blood pump membranes was first reported in 1975 in a TAH implanted for 30 days in a calf model [68]. Although sheep are considered the gold standard for studying calcification in heart valve research, many early studies on calcification in animal-derived and polymeric materials used in LVADs and TAHs were performed in calves [69,70]. Over recent decades, LVAD designs have shifted from positive displacement to rotary pumps, replacing flexible membranes with rigid components. Because calcification typically occurs in compliant materials, this design evolution has markedly reduced calcification risk, reducing the need for dedicated large animal calcification testing of blood pumps. Recently, however, new blood pump designs have reintroduced flexible components [44,71,72]. This may reintroduce calcification risks in flexible blood-contacting surfaces. Consequently, renewed attention to in vivo large animal studies to evaluate calcification in these devices is warranted.

## 5. Interspecies Variability in Thrombogenicity Assessment of Cardiac Devices

Thrombogenicity refers to the tendency of a blood-contacting implant to promote thrombus formation. Thrombus development is a multifactorial process that can be described by the three principal components of Virchow’s triad: endothelial injury, hemodynamic disturbances, and a hypercoagulable state. These factors induce platelet responses and coagulation system activation [73,74,75]. In the context of mechanical cardiac implants, thrombogenicity is typically driven by device surface properties, hemodynamic factors such as stasis and non-physiological shear stress, and host hemostatic responses which disrupt normal blood homeostasis [74,76,77].

The minimization of thrombogenicity is essential for safe clinical implementation of mechanical cardiac implants, given that elevated thrombogenic potential is associated with severe thromboembolic complications, including stroke, device malfunction, and failure. This requires preclinical evaluation of thrombogenicity in relevant large animal models. Sheep, goat, pigs and calves are used for assessing cardiac device thrombogenicity. However, predictive reliability of these assessments for clinical application is limited by substantial interspecies differences. An illustrative example is the Medtronic Parallel valve, which proceeded to clinical trials in the 1990s after showing no signs of thrombosis in sheep studies. Despite, these clinical trials were later discontinued due to excessive thrombus formation in patients [78].

Mizuno et al. compared clotting parameters of goat, pig and calf blood to human values [79]. Calves showed significantly prolonged whole-blood activated clotting times, as well as significantly longer extrinsic and intrinsic clotting times, compared with humans. In contrast, goats and pigs exhibited extrinsic and intrinsic clotting times that did not differ statistically from human values. These results may therefore discourage the use of calves as a model for assessing coagulation behavior in response to cardiac implants. A systematic review by Staelens et al. found that sheep have intrinsic pathway activity comparable to humans, reflected by similar activated partial thromboplastin times (aPTT), whereas extrinsic pathway activity was lower, as indicated by prolonged prothrombin times (PT) [80].

**Figure 3:**
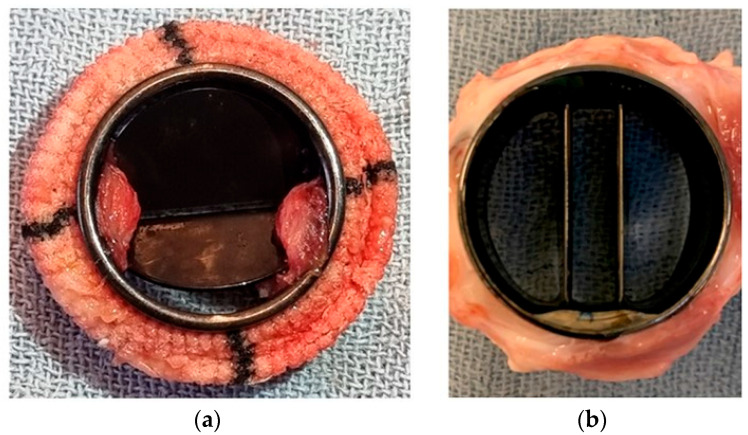
Macroscopic appearance of explanted mechanical valves in the pulmonary position. From Van Hecke et al. [81], used with permission from the authors: (**a**) Valve explanted from a pig model showing visible thrombus formation. (**b**) Valve explanted from a sheep model showing no visible thrombus formation.

Van Hecke et al. directly compared the thrombogenic potential of mechanical heart valves between sheep and pigs, by implanting bileaflet mechanical valves in the pulmonary position without anticoagulation [81]. Notably, all pigs sacrificed between 14 and 38 days had visible thrombi on the valve surface, whereas none of the sheep sacrificed between 71 and 155 days showed signs of thrombosis (Figure 3). In line with these findings, Goodman et al. observed attenuated platelet responses in sheep compared to pigs and humans in vitro [82]. The interaction of sheep, pig and human platelets was tested on various materials. They found that ovine platelets neither attach, spread, nor form thrombi like human platelets, while porcine platelets more closely resembled human biological responses. The increased thrombogenic tendency of pigs compared to humans might originate from their higher platelet count and stronger platelet aggregation [80].

Although sheep remain the gold standard for preclinical heart valve testing, these findings question their physiological relevance for thrombogenicity assessment and emphasize the limitations in translation of preclinical results to humans. Instead, pigs may serve as a more suitable model.

For a true worst-case scenario with respect to thrombogenicity, animal studies should ideally be conducted without antiplatelet or anticoagulant drugs postoperatively. However, when anticoagulation is deemed necessary, for example in studies in which thrombogenicity is not the primary outcome, researchers must recognize that the effectiveness of anticoagulant drugs differ substantially between humans and animals. Understanding these interspecies variations in coagulation biology is important to optimize peri- and postoperative anticoagulation protocols. For example, acetylsalicylic acid has demonstrated limited efficacy to inhibit ovine platelet aggregation [83]. As an alternative, clopidogrel showed modest efficacy and heparin and warfarin are proven to be safe and effective in sheep and goats [84].

## 6. Hemodynamic Assessment of Cardiac Devices in Large Animal Models

Prosthetic heart valves, LVADs, and TAHs all directly affect the pump function and flow dynamics of the native heart and vasculature. Suboptimal interactions between these devices and blood circulation can profoundly affect patient outcomes and quality of life. In vitro evaluations using pulse duplicator systems and mock circulatory loops provide an essential first step for systematically characterizing the hemodynamic performance of valves, LVADs, and TAHs under controlled conditions. These platforms are indispensable for comparing design iterations, optimizing operating parameters, and meeting regulatory requirements due to their measurement accuracy and experimental standardization. However, in vitro assessments cannot fully replicate anatomical constraints or dynamic boundary conditions. Moreover, post-implantation host responses—such as tissue overgrowth, calcification, and vascular adaptation—cannot be simulated in vitro, yet strongly influence hemodynamic performance. Large animal models therefore serve as a critical complement to in vitro studies, enabling assessment of device performance under realistic anatomical, physiological, and biological conditions.

Chronic evaluation of prosthetic heart valves in large animal models enables longitudinal monitoring of calcification progression and thrombus formation while also providing a platform to assess how these processes affect hemodynamic performance. For proper translatability of hemodynamic performance, the animal models used should preferably have a circulatory system in which the valve is exposed to hemodynamic parameters similar to those in humans. Table 1 summarizes the body weight and resting cardiac index values reported by Lin et al. and Li et al. [8,9]. Based on these data, we calculated corresponding cardiac output ranges. For comparison, representative human resting values are also included in the table [85]. Interestingly, humans have a much lower cardiac index compared to quadrupeds.

Sheep are most commonly used for chronic prosthetic valve experiments. The cardiac output of adult sheep ranges between 4.9 and 10.8 L/min, making this species well suited for evaluating hemodynamic performance. As noted previously, pigs are considered appropriate models for evaluation of thrombogenic tendency of valves. However, from a hemodynamic perspective, adult pigs exhibit a cardiac output that greatly exceeds that of humans, reaching values up to 21.8 L/min. Goats and calves are not typically employed for prosthetic heart valve assessment.

**Figure 4:**
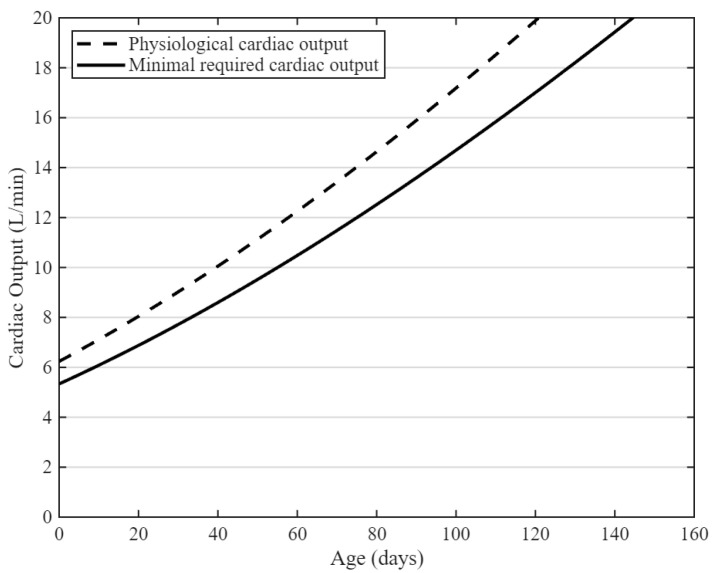
Physiological and minimal required cardiac output for calves.

In vivo evaluation of LVADs and TAHs requires animal models with a cardiac output comparable to humans. This is specifically relevant for TAHs, which completely replace the native heart’s function. To ensure proper perfusion, the animal model should not have cardiac output requirements greater than the devices’ maximum capacity. In practice, this poses a challenge because, due to the previously mentioned spatial requirements, TAHs often only fit within the thoracic cavity of a calf. However, the continuous somatic growth of these animals causes a physiological mismatch between the device’s maximum cardiac output capacity and the calves’ minimal perfusion requirements. The impact of this growth-related mismatch has been highlighted in prior TAH studies, where a markedly higher complication rate was observed in calf experiments compared to human trials using the same device [23]. These complications were, in part, attributed to the increasing disparity between the animal’s physiological requirements and the device’s functional capacity over time. The normal resting cardiac index of calves is 152 mL/min/kg while minimal cardiac index required to maintain physiologically stable conditions ranges from 120 to 140 mL/min/kg [86,87]. Assuming a relatively constant cardiac index during growth, cardiac output increases proportionally with body weight. Combined with the calf growth curves shown in Figure 1, this results in the projected minimal cardiac output requirements depicted in Figure 4. Figure 4 demonstrates a rapid rise in circulatory demand during early development. For example, by three months of age, the required cardiac output exceeds 13 L/min, surpassing the capacity of most TAH systems.

In contrast to TAHs, LVADs are implanted to augment the native heart’s function. Thus, assuming the absence of heart failure, animal survival is not dependent on the LVAD’s output. Long-term follow-up in calf and pig models is therefore feasible despite their relatively high circulatory demands. In clinical practice, LVADs are implanted in patients with severely impaired cardiac function. For proper translation of LVAD research from animal models to clinical practice, it is often necessary to create heart failure models that reflect the impaired cardiac function of patients receiving LVAD therapy [88]. Numerous heart failure induction methods have been described, but ischemic models of myocardial infarction remain the most widely used in LVAD evaluation. The two principal approaches to inducing ischemic cardiomyopathy are catheter-based coronary embolization and coronary ligation. Catheter-based coronary embolization offers several advantages: it is minimally invasive, preserves an undisturbed chest cavity for subsequent LVAD implantation, enables targeted injury based on the specific coronary vessel embolized, and has been successfully employed in sheep, goats, pigs and calves [88,89]. Coronary ligation, another established method, produces well-demarcated infarcts and effectively models acute post myocardial infarction physiology. However, it requires a thoracotomy or sternotomy, which increases perioperative recovery demands and disrupts the mediastinal environment needed for later device implantation [88].

LVADs and TAHs should respond dynamically to the patient’s circulatory system, which is continuously modulated by posture, physical activity, and disease states. Experimental protocols have been developed to evaluate the hemodynamic adaptability of these devices in both acute and chronic animal studies. Acute animal experiments allow for assessment of device responsiveness under controlled conditions. In these studies, specific hemodynamic states can be deliberately induced, enabling detailed monitoring of device performance. Two key characteristics of blood pumps commonly evaluated in animal models are preload and afterload sensitivity. Preload sensitivity refers to the device’s ability to increase pump output in response to elevated venous return, analogous to the Frank-Starling mechanism in the native heart. Preload can be modulated through saline infusion or venous drainage into a cardiopulmonary bypass reservoir [90]. The use of a pneumatic occluder in the vena cava to regulate venous return has also been reported in the literature [91]. Afterload sensitivity, which describes the device’s response to changes in vascular resistance, can be assessed by rapidly altering systemic or pulmonary vascular resistance. Vascular resistance can be increased by partial clamping of the aorta or pulmonary artery, or by administering vasopressors [92]. A decrease in vascular resistance can be achieved by administering vasodilators [90].

Chronic animal experiments offer fewer opportunities for isolated manipulation of venous return and vascular resistance. Instead, physiological activity-related cascades are typically induced through postural changes and exercise protocols, such as treadmill-based testing [93,94,95,96]. These protocols provide physiologically relevant means of assessing device adaptability under dynamic loading conditions.

Evaluation of hemodynamic performance requires monitoring parameters such as atrial and systemic pressures, as well as cardiac output. Pressure measurements are most commonly obtained with fluid-filled pressure lines or catheter-based transducers, with sensor placement in the aorta, pulmonary artery, atria or ventricles [97,98]. Flow measurements are typically performed using ultrasonic flow probes, positioned around the pulmonary artery, ascending aorta, LVAD outflow graft, or combinations thereof, depending on experimental objectives [72,97,99]. During acute experiments, these measurements are relatively straightforward to perform, while monitoring these parameters in chronic animal studies is more challenging. Some circulatory support devices feature integrated sensors for real-time measurement or estimation of cardiac output, atrial pressures, and arterial pressures [23]. Where such built-in sensing capabilities are absent, chronic instrumentation must be employed for long-term monitoring.

## 7. Advancing Ethics Through Implementation of the 3Rs Principle

In recent decades, increasing attention has been directed towards reducing animal use and minimizing animal suffering in scientific research. This effort is guided by the 3Rs—Replacement, Reduction, and Refinement—introduced by Russell and Burch in the 1950s, and now embedded into European legislation on the protection of animals used for scientific purposes [100].

The Replacement principle promotes the use of non-animal approaches, such as in silico and in vitro models, to replace the use of animals in preclinical research. Recent technological advances, among which physics-based and artificial intelligence-driven computational models for medical device evaluation, as well as sophisticated in vitro systems, have significantly expanded the potential of replacement strategies [101,102]. For example, mock circulatory systems have evolved into hybrid platforms in which physical hydraulic components are combined with computational models, incorporating more complex physiological control mechanisms, such as baroreflex and hormonal responses [103]. In addition, in vitro accelerated calcification models, in which valve tissue is exposed to calcifying solutions under controlled conditions, can be used to assess the susceptibility of synthetic materials to mineral deposition [51]. Thrombogenicity can be assessed using dynamic blood-material interaction studies, in which platelet activation and clot formation are evaluated under controlled flow conditions [104]. Although these methods may not ultimately eliminate the need for animal models, they allow in vivo experiments to be reserved for later stages of device development.

Reduction is the principle of minimizing the number of animals used in research and can be achieved through several complementary strategies. Proper statistical design and power analysis are commonly considered the most effective approaches to reducing sample sizes. Unlike small animal experiments, large animal studies are often qualitative rather than quantitative and therefore typically require relatively small sample sizes. In addition, wastage of animals due to inconclusive or irrelevant outcomes and excess mortality should be avoided to prevent repetition of experiments with additional animals. This can be achieved through careful selection of the most appropriate animal model for each specific research objective, ensuring that meaningful results are obtained. Mortality may be reduced by conducting cadaver and pilot studies for early assessment and optimization of experimental protocols, and to accelerate the procedural learning curve. Additionally, reusing animals from chronic experiments in subsequent acute studies can limit overall animal use, considering that acute procedures impose minimal additional burden.

Refinement focuses on improving animal welfare by minimizing pain and distress and promoting positive affective state. This can be achieved by providing housing conditions that encourage natural, species-specific behaviors, applying appropriate anesthesia and analgesia protocols, and defining clear humane endpoints in advance. In addition, training can be implemented to promote cooperation and familiarity with procedures, thereby reducing stress and discomfort [105]. For example, when conducting LVAD or TAH research that requires animals to carry externally mounted batteries, tolerance to these conditions can be established through pre-implantation training.

## 8. Discussion and Conclusions

Large animal studies play a central role in the safe and effective translation of medical devices from conceptual design to clinical application, as they enable device evaluation within the physiological complexity of a living organism under biological, structural, and mechanical conditions. However, substantial differences between humans and large animal models require careful consideration when selecting an appropriate model. This is essential not only to ensure scientifically valid results but also, from an ethical perspective, to maximize the scientific value obtained from each animal study. At present, no general consensus exists regarding optimal animal model selection. We therefore aimed to provide guidance on large animal model selection—specifically sheep, goats, calves, and pigs—and experimental design for the evaluation of mechanical cardiac implants. Our findings are organized according to relevant research objectives in this context, including calcification, thrombogenicity, and hemodynamic performance and are summarized in Table 2.

Large animal experiments are often poorly reported, undermining reproducibility. Essential details such as animal breed, age, sex, model selection rationale, and observed complications during or after implantation procedures are frequently missing [106]. For example, endocarditis, a severe post-implantation infection associated with cardiac devices [60,107]. While this complication can significantly impair device function, increase mortality, and necessitate early termination of experiments, the occurrence of endocarditis is often not well-reported. The ARRIVE guidelines provide recommendations to improve planning, design, and reporting of in vivo research, enhancing publication quality and ultimately supporting better clinical translation [108].

The paper is designed as a narrative review rather than a systematic analysis, with findings presented qualitatively rather than quantitatively. Although it does not aim to cover all available literature on the subject, this format enables a comprehensive and practical overview of key insights essential to large animal experimentation in the evaluation of mechanical cardiac implants.

In addition, the scope of this review is limited to general animal model characteristics and features related to anatomy, calcification, thrombogenicity, and hemodynamic performance. Depending on the type of implant, however, additional characteristics and interspecies differences may play an important role in animal model selection. This is particularly relevant for tissue-engineered heart valves. Tissue engineering involves complex and not yet fully understood biological processes that may vary significantly between species, limiting direct clinical translatability [109]. Therefore, the considerations presented in this review may not be sufficient to guide animal model selection for tissue-engineering applications.

In conclusion, appropriate selection of large animal models is essential for effective and translationally relevant preclinical evaluation of mechanical cardiac devices. However, model selection is still often guided by convention rather than evidence, and standardized reporting remains limited. Greater adherence to reporting frameworks such as the ARRIVE guidelines could improve transparency, reproducibility, and the overall quality of preclinical animal research. Each species presents distinct anatomical and physiological characteristics that influence device performance. Sheep are preferred for calcification studies and pigs for thrombogenicity testing. Sheep or goats are most suitable for hemodynamic evaluation due to their human-like cardiovascular parameters. However, for TAH evaluation, calves are typically used because of spatial requirements, despite growth-related limitations. Overall, we conclude that there is no single animal model capable of evaluating all aspects of a device for translational purposes. Instead, different animal models provide distinct advantages depending on the outcomes of interest.

## Figures and Tables

**Table 1:** Hemodynamic parameters of sheep, goats, pigs, calves and humans.

Animal	Weight (kg)	Cardiac Index (mL/min/kg)	Cardiac Output (mL/min)
Sheep (adult)	40.8–90.7	119 (SD 41)	4.9–10.8
Goat (adult)	40.8–81.6	136 (SD 30)	5.6–11.1
Pig	up to 150	145 (SD 27)	up to 21.8
Calf	20–350	152 (SD 46)	3.0–53.2
Human (adult)	68 (SD 14)	67	4.58 (SD 1.12)

**Table 2:** Overview of large animal models for mechanical cardiac implant evaluation. Suitability for thrombogenicity and calcification assessment is indicated using a qualitative scale: − − (not suitable), − (not ideal), and + (suitable).

Animal	Typical Weight (kg)	Cardiac Output (L/min)	Key Anatomical Characteristics	Typical Device Applications	Thrombo- Genicity	Calcification Tendency	Main Advantages	Key Limitations
Sheep	40.8–90.7	4.9–10.8	Short ascending aorta; valve anatomy similar to humans	Prosthetic valves; LVADs; (small) TAHs	− (attenuated platelet response)	+ (especially juvenile sheep)	Well-established; manageable; sensitive to calcification; stable adult size	Underestimates thrombogenicity
Goat	40.8–81.6	5.6–11.1	Similar to sheep; smaller thoracic cavity	LVADs; (small) TAHs	Unclear	Unclear	Stable adult size	Limited data
Pig	Up to ~150	Up to 21.8	Human-like coronary anatomy	Acute studies; thrombogenicity; training	+ (human-like platelets)	Unclear	Best thrombogenicity model	Growth and behavior limit chronic studies
Calf	~20–350	3.0–53.2	Large thoracic cavity; short ascending aorta	TAHs; LVADs	− − (prolonged clotting times)	Unclear	Only model for large TAHs	Growth limits chronic studies

## Data Availability

No new data were created or analyzed in this study. Data sharing is not applicable to this article.
